# Structure based docking and biological evaluation towards exploring potential anti-cancerous and apoptotic activity of 6-Gingerol against human prostate carcinoma cells

**DOI:** 10.1186/s12906-023-04269-1

**Published:** 2024-01-02

**Authors:** Habiba Khan, Iqbal Azad, Zeeshan Arif, Shama Parveen, Saurabh Kumar, Juhi Rais, Jamal Akhtar Ansari, Malik Nasibullah, Sudhir Kumar, Md Arshad

**Affiliations:** 1 Department of Zoology, University of Lucknow, 226007 Lucknow, U.P India; 2 Department of Chemistry, Integral University, Kursi Road, 226026 Lucknow, U.P India; 3 Computational Toxicology Facility, Toxicoinformatics and Industrial Research, CSIR-Indian Institute of Toxicology Research, 31 Mahatma Gandhi Marg, 226001 Lucknow, U. P India; 4 Academy of Scientific & Innovative Research (AcSIR), 201002 Ghaziabad, India; 5 Department of Nuclear Medicine, Sanjay Gandhi Postgraduate Institute of Medical Sciences, 226014 Lucknow, India; 6 Department of Zoology, Aligarh Muslim University, 202002 Aligarh, India

**Keywords:** Prostate cancer, Androgen receptor, 6-Gingerol, Molecular docking, Anticancer

## Abstract

**Background:**

6-Gingerol (6-G) is the primary active phytocomponent of ginger and has been shown to regulate multiple targets against cancer and its treatment. Androgen receptors (ARs) remain critical in the progression of prostate cancer (PCa). This study focuses on investigating 6-G as a promising anti-cancerous agent that inhibits AR activity significantly.

**Methods:**

In this study, molecular docking simulation was done to investigate the binding affinity of 6-G and control drug Bicalutamide (BT) against oncogenic AR and tumor suppressor estrogen receptor β (ERβ). The crystal structure of AR and ERβ was retrieved from Protein Data Bank (PDB) and docked with 3D Pubchem structures of 6-G using iGEMDOCK and AutoDock. Further *in vitro* study was done to evaluate the antioxidant, anti-cancerous, apoptotic, and wound healing potential of 6-G.

**Results:**

The result displays that 6-G shows good binding affinity with AR and ERβ. Condensation of the nucleus, change in mitochondrial membrane potential (MMP) and the ability to induce reactive oxygen species (ROS) were done in human PCa PC-3 cells. Results from the MTT assay demonstrated that 6-G and control drug BT showed significant (*p* < 0.01) dose and time dependent inhibition of human PCa PC-3 cells. 6-G increased the ROS generation intracellularly and decreased the MMP, and cell migration in treated PCa PC-3 cells. 6-G treated cells showed fragmented, condensed chromatin and nuclear apoptotic bodies.

**Conclusions:**

Thus, this study validates 6-G as a potential drug candidate against human PCa. However, further study of the anticancer potency of 6-G has to be done before its use for PCa treatment.

## Introduction

Prostate cancer (PCa) has become the third leading cause of cancer mortality among elderly men worldwide. Androgen receptor (AR) plays a critical role in the initiation, invasion and PCa progression. The AR gene is amplified, and gain-of-function mutations, oxidative stress, and inflammation are some of the factors that cause AR to express [1]. Previous studies suggested AR signalling regulated expression of several DDR genes (*BRCA1, BRCA2*, and *ATM* ) in late PCa [36]. Mutation in DDR genes has been shown to promote cell survival, cell cycle progression, DNA damage repair in cancer cells [37, 38]. Several studies reported the role of estrogen receptors (ERα, ERβ) in the progression and metastasis of PCa [2]. A recent study suggests that among the Estrogen receptors, the subfamily proteins ERα is found to play an oncogenic role whereas ERβ exerts a tumour-suppressing role in PCa [3]. Major therapeutic options for the treatment of PCa include medical castration of androgen and/or use of anti-androgens and/or AR antagonists drugs (Cyproterone acetate, Enzalutamide, Bicalutamide) [4]. Most patients respond to these treatment options in the initial years but later they develop several side effects and, in many cases, cancer reoccurs and progresses to the metastatic stage reducing their therapeutic potential [5]. Some of the most common side effects are urine incontinency, dysfunction of erectile, bowel disorders, rectal discomfort, and in some cases cardiotoxicity, gynecomastia, and memory loss [6, 7]. Altogether there is a need to develop safer and more effective chemotherapeutic agents for the prevention and treatment of PCa. In the past few years, dietary phytochemicals have attracted much attention from many researchers due to their various pharmacological and biological properties [8, 9]. A bulk of research has been done on dietary phytochemicals for their anti-proliferative, anti-angiogenic, and anti-metastatic effects and their ability to halt the cell cycle and induce apoptosis in many cancer types [10. The anti-inflammatory and antioxidant activity of phytochemicals targeting AR directed cell survival, progression of cell cycle, repair of DNA damage and their ability to modulate several molecular signaling pathways can be promising candidates in the treatment and prevention of PCa [11].

Polyphenolic category of phytochemicals are extensively found within the plant kingdom contain several phenol structural units [12]. Polyphenols, such as flavanols have structural similarities to testosterone thereby they may bind to ARs with high affinity which could lead to safer and more effective options for PCa treatment [10]. *Zingiber officinale Roscoe* commonly known as ginger has 6-Gingerol (6-G) as the main active flavonoid and is known to be responsible for most of the pharmacological activities of ginger [13]. 6-G works by altering various biological mechanisms that control apoptosis, cell cycle regulation, and cytotoxic action [14, 15]. 6-G has been demonstrated to regulate pro-apoptotic proteins Bax, Bid, TNFa, and genes producing cytochrome c while downregulating anti-apoptotic proteins c-FLIP, Bcl-2, and XIAP [16]. Consequently, understanding how 6-G interacts with other substances can help with their prospective use in PCa treatment. Molecular docking is one of these often-employed techniques since the interaction of the protein and the ligand is crucial to the design of drugs with a structure [17]. Molecular docking accurately predicts the conformation of small-molecule ligands inside the appropriate target binding site. Structure based docking of 6-gingerol on androgen as well as estrogen receptors is not been well studied. Hence, molecular docking has been utilized to investigate efficacy and how 6-G interact with ERβ and AR. To investigate 6-G anti-mutagenic and anti-carcinogenic capabilities against late PCa, additional *in vitro* research will be conducted. Thus, this study is aimed to evaluate the role of 6-G on human PCa PC-3 cells and their affinity to bind with ERβ and AR. Therefore, the finding of this study will elucidate the binding affinity, efficacy and molecular interaction of 6-G against nuclear receptors, which will provide new insight for its use in structure-based drug design. Further, this study will illustrate the cellular and molecular mechanism of proliferation and apoptosis *viz.* ROS, nuclear dye diamidino-2-phenylindole dihydrochloride (DAPI), acridine orange/propidium iodide (AO/PI), mitochondrial membrane potential (MMP), cell cycle arrest and wound healing assay suggesting its AR mediated effect on DDR genes. Therefore, AR regulate DDR factors and these results opens new strategies to improve diagnostic, prognostic and therapeutic approaches. However, further studies are needed to validate the results and other processes in PCa.

## Materials and methods

### Computational details

#### Molecular docking simulation

**Fig. 1:**
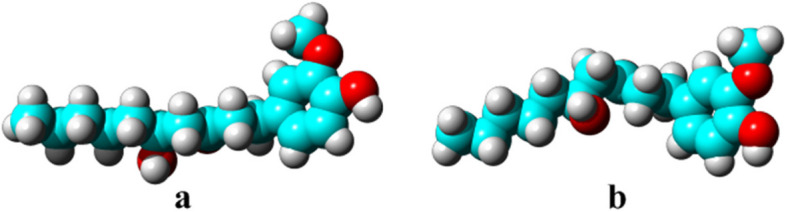
3D structure of 6-Gingerol (**a**) Non-optimized (**b**) Optimized

iGEMDOCK v2.1 was performed for docking evaluation, to find the binding interaction of ligands at the active site of ERβ and AR protein [18]. The crystal structure of human ERβ and AR protein with the PDB IDs: 1U9E and 2AM9 were obtained from Protein Data Bank (PDB, http://www.pdb.org). Protein structure refinement including cleaning, minimization, and structure optimization was completed with the help of the 3Drefine server. It provides a complete package of outcomes that contains the subsequent information like five various refined protein models and the potential energies of all the models after energy minimization [19]. For defining, outlining, and calculating the geometrical as well as topological parameters of receptor proteins, the CASTp v3.0 (Computed Atlas of Surface Topography of proteins) server was used. The updated version of CASTp delivers the information such as routes of the adverse pocket volumes, cavities and canals, topographic structures, better visualization, and additional information like efficient sites, modified sites, and other important observations. Radius probe 1.4 Å was used to determine the active site of the ligand [20]. Biovia Discovery Studio (BDS) Visualizer v2017R2 was used to visualize the 3D structure of protein and docking interaction profile. The crystal structure of 6-G was downloaded from PubChem (https://pubchem.ncbi.nlm.nih.gov/). Avogadro v1.2.0 was further used to additionally optimize the structure of ligands with the help of MMFF94 (Merck Molecular Force Field) displayed in Fig. 1. The docking results are obtained in the form of total energy, a combination of the hydrogen bond, van der Waals, and electrostatic interaction energies. For the docking validation redocking as well as docking with AutoDock v4.2 and MGL Tools v1.5.6 were also used.

#### IC_50_ calculation

To understand the actual tentative activity of the recognized lead compound against the receptor proteins (ERβ and AR), the half-maximal inhibitory concentration (IC_50_) value was evaluated with the help of AutoDock v4.2 and MGL Tools v1.5.6. It is a scale of the strength of a molecule in inhibiting a specific receptor protein [21]. The grid box was prepared to cover the active site domain of ERβ and AR along with grid coordinates as well as grid size in xyz direction. The value of the grid coordinate is set at 19.42, 34.0, and 38.07 for ERβ, while at 20.38, 5.33, and 11.21 for AR. On the other hand, the value of grid size was set at 26×26×26 Å in xyz direction for both the receptor proteins with the 1.0 Å grid spacing. To find the best docking conformer, the Lamarckian genetic algorithm (LGA) along with default docking parameters and atomic solvation parameters 126 Å in xyz direction were used. The LGA cluster study was performed to obtain the binding energy of ten docking complexes with their IC_50_ values. The best docking complexes were obtained in terms of lowest binding energy and IC_50_ value. The validation of obtained results, and redocking was performed twice.

#### DFT calculations

The electron transport potential and the qualitative estimation of electronic properties were evaluated with the help of density functional theory (DFT). Gaussian 9W and Gauss View v6.0 were used to estimate the DFT calculation [39, 40]. In the ground state (gaseous and solvent phases), the molecular structure of 6-G was optimized using DFT with the B3LYP method with 6–31 + G(d), 6-31G(d,p), 6-311G(d,p), 6-311 + + G(d,p), and 6-311++(2d,2p) basis sets [39–41]. Using the B3LYP approach, time-dependent DFT (TD-DFT) has been utilized to compute the energies and strengths of the lowest-energy spin-permitted electronic excitations in the solvent and vacuum phases using the polarized continuum model (PCM) [42]. DFT/B3LYP/6-311G++(2d,2p) level of theory used to evaluate the frontier molecular orbitals (FMOs) such as the HOMO (highest occupied molecular orbital) and LUMO (lowest unoccupied molecular orbital) are well-known and deliver relevant data about electron density clouds of the molecule [43]. The non-bonding molecular orbital (NBMO) is referred to as HOMO, is favored by electrophilic attacks whereas π molecular orbital is referred to as the LUMO, favored by nucleophilic attacks [21]. The energy of HOMO and LUMO is the most common quantum chemical parameter (QCP) that denotes the ability of electrons to donate and accept. The higher energy value of HOMO supports the ability of electron donation. The energy gap (E_GAP_) is referred to as the energy difference between HOMO and LUMO. It is another significant parameter of QCP, that is also used to evaluate the reactivity of chemical species such as antibacterial activity. The lower value of E_GAP_ represents the improved antibacterial effect, thus the lowest value of E_GAP_ increases the value of reactivity of chemical species, which ultimately improves the electron-donating proficiency.

#### Molecular descriptor calculations

Lipinski’s rule of five (Ro5) is one of the most broadly used rules for the evaluation of oral bioavailability of chemical species obtained with the help of the Molinspiration server 2020 (https://www.molinspiration.com). Almost orally administrated drugs obey Ro5, such as 5 ≥ H-bond donors (nON), 5 ≥ LogP, and 500 Da ≥ molecular weight (MW). 10 ≥ H-bond acceptors (nNHOH) and 10 ≥ rotatable bonds (RB) [22]. The parameter of the Ro5 offers broad information about the chemical species as a drug-likeness. MW is typically associated with the absorption of chemical species at the surface of the intestinal epithelium, higher MW value support lower absorption. On the other hand, the value of LogP (octanol-water partition coefficient) is related to the permeation power across a biological membrane, thus higher LogP represents poor permeation power.

#### Bioactivity radar prediction

The bioavailability radar is another new graphical representation and calculation of the drug-likeness of a chemical species obtained from six pharmacokinetic parameters such as LIPO (Lipophilicity): -0.7 < XLOGP3 < + 5.0; SIZE: 150g/mol < MW < 500g/mol; POLAR (Polarity): 20Å2 < TPSA < 130 Å^2^; INSOLU (Insolubility): 0 < LogS (ESOL) < 6; INSATU (Instauration): 0.25 < Fraction Csp3 < 1; FLEX (Flexibility): 0 < Num, rotatable bonds < 9. In the graph the pink zone signifies the best range for the respective parameter, thus it is the most suitable pharmacokinetic space for oral bioavailability [23].

#### ADMET properties calculation

It is gradually more noticeable that ADMET (absorption, distribution, metabolism, excretion and toxicity) parameters are a significant figure for the progress of promising novel drugs. The failed ADMET features can be declined to display drug-like properties in the later phase of drug development admetSAR (ADMET structure-activity relationship) is extensively utilized in chemical and medicinal areas, it is a regularly updated open server [24]. AdmetSAR is frequently managing ADMET-related information from the available literature. In admetSAR v1.0, over 210,000 interpreted data opinions for 96,000 novel molecules, 27 computer-aided models and 45 varieties of ADMET-related information thoroughly manage. In the updated version of admetSAR 2.0, the number of models is increased from 27 to 47 for drug development and environmental hazard calculation. In updated version additionally introduces a new segment termed as ADMETopt for hit optimization that is built on projected ADMET parameters [25]. *In silico* ADMET evaluation boosts the protocol of drug design and development such as HIA (human intestinal absorption), Caco-2 permeability and Log Kp (skin permeation) models can calculate the oral, intestinal and transdermal absorption. Plasma protein binding (PPB), Blood-brain barrier (BBB) penetration, Estrogen receptor binding (ERB), Androgen receptor binding (ARB), P-glycoprotein (P-gp) substrate and P-glycoprotein (P-gp) inhibitor are variable parameters associated with the distribution [26]. PPB model provides information on disposition and efficiency, BBB penetration can offer information on chemical species to cross the central nervous system (CNS), and ERB and ARB models explain the inhibition of ER and AR. P-gp substrates and inhibitor models usually give information about the transport of xenobiotics into the intestinal lumen. *In silico* toxicity calculation will have great significance in the primal stage of drug development, subsequently, 30% of drug applicants failed due to these limitations [24].

#### BOILED Egg (Brain Or IntestinaL EstimateD) model

HIA and BBB are the significant pharmacokinetic parameter perilous to be estimated throughout the drug development approach. The BOILED egg model was recently created by employing Egan's egg to test the HIA and BBB crossover capacity. It is accurately predicting the pharmacokinetic parameter in the form of a graph. The BOILED egg model was obtained based on the value of HIA, BBB penetration, lipophilicity and polarity [27].

#### Bioactivity score

In finding a probable hit compound, it is critical to identify the most suitable receptor protein. Thus, preliminary chemical species are subjected to evaluate the bioactivity against the available receptor protein such as G-protein coupled receptor (GPCR) ligand, ion channel modulator (ICM), kinase inhibitor (KI), nuclear receptor ligand (NRL), Protease inhibitor (PI) and Enzyme inhibitor (EI), it is easily obtained in the form of bioactivity score. Molinspiration is an open source to check the bioactivity score [28].

### Evaluation of anticancer activity

#### Reagents

Roswell Park Memorial Institute (RPMI) 1640 medium, fetal bovine serum (FBS), MTT (3-(4,5-dimethylthiazol-2-yl)-2,5-diphenyltetrazolium bromide) dye, 4′,6-diamidino-2-phenylindole (DAPI), 2′,7′-Dichlorofluorescein diacetate (DCFH-DA), MitoTracker Red CMXRos, Acridine Orange (AO) and Propidium Iodide (PI) were purchased from Himedia, India for anticancer activity assessment. Calbiochem supplied the dimethyl sulfoxide (DMSO) (CA, USA). A Milli-Q system was used to create ultrapure deionized water (Millipore, Bedford, MA, USA). All of the chemicals used in cell culture experiments were of excellent quality.

#### hytochemical and control drug

Phytochemical 6-Gingerol was purchased from Sigma-Aldrich and Bicalutamide was used as a control drug purchased from pharmacy Lucknow, India.

#### Cell line

PCa cell line PC-3 was obtained from the National cell repository-National Centre for Cell Sciences (NCCS), Pune, India.

#### Cell culture

PC-3, a human PCa cell line, was grown in RPMI 1640 media supplemented with 10% (v/v) FBS, 0.1mM non-essential amino acids, 2.0 mM L-glutamine, 1.0mM sodium pyruvate, 1.5 g/l NaHCO3, and 1% antibiotic solutions. The PC-3 cells were grown in humidified air at a temperature of 37°C and 5% CO2.

#### Morphological examination

The phase contrast inverted microscope was used to study the dose-dependent effect of compound 6-G and control drug BT on the cellular morphology of PC-3 cells and 6-G on the normal HaCaT cells (Nikon ECLIPSE Ti-S, Japan). The PC-3 and HaCaT cells were seeded at a density of 2x10^4^ cells per well in a 96-well plate. The cells were cultivated overnight before being treated for 24 hours with 6-G in the 40–120 µM range and the control medication BT in the 5–25 µM range. After 24 hours of exposure, the cellular morphology of treated and control cells was examined as previously described [29].

#### MTT cell viability test

Cell viability was determined using a standard colorimetric assay based on MTT (CellTiter 96 AQeous, Promega, Madison, WI, USA) [30]. The enzymatic degradation of MTT dye to purple formazan crystals is the basis for this colorimetric assay. Selective dosages of 6-G (40, 60, 80, 100, and 120 µM) and Bicalutamide (5, 10, 15, 20, and 25 µM) were applied to grown PC-3 cells. Selective dosages of 6-G (40, 60, 80, 100, and 120 µM) was also applied on normal HaCaT cells. These plates will be incubated for 24 hours at 37°C. MTT reagent (10µl/well) was added to each well after 24 hours, and plates were incubated for 3 hours at 37°C in an incubator until purple-colored formazan crystals appeared. Media was removed and to each well 100 µl of DMSO was added and formazan crystals were dissolved. The plate was maintained at 37^o^C for another 10 minutes. A microplate reader was used (BIORAD-680) to measure the absorbance at 540 nm. The relative percent viability of cells was computed using the formula:

$$\mathrm{Cell}\;\mathrm{viability}\;\mathrm{as}\;\mathrm a\;\mathrm{percentage}\;=\;\left[\left(\mathrm{OD}\;\mathrm{of}\;\mathrm{treatment}\right)/\left(\mathrm{OD}\;\mathrm{of}\;\mathrm{control}\right)\right]\;\times\;100$$

#### Reactive oxygen species (ROS) activity

After treating the PC-3 cells with different doses of 6-G, microscopic fluorescence imaging was performed to estimate ROS. PC-3 cells (2x104 cells per well) were cultivated in 96-well culture plates and treated with three effective dosages of 6-G: 60, 80, 100, and 120 µM. Cells were then treated for 30 minutes at 37^o^C to 10 mM of DCFH-DA which act as a fluorescent agent. The mixture was further aspirated in each well and rinsed with PBS, and pictures were captured using an fluorescence inverted microscope (Nikon ECLIPSE Ti-S, Japan). In a 96-well black-bottomed culture plate, cells (2x10^4^ cells per well) were planted and treated for quantitative fluorescence intensity analysis. After exposure, cells were treated for 30 minutes at 37°C with DCFH-DA (10 mM). In each well, the reaction mixture was withdrawn and rinsed with PBS (200 ml). The plate was shaken in the dark for 10 minutes at room temperature (RT). Image J software was used to assess relative fluorescence intensity in a SYNERGY-H1 multiwell plate reader (Bio-Tek, Winooski, VT) at excitation and emission wavelengths of 485 and 528 nm, respectively. All of the values were reported as a percentage of the control's fluorescence intensity. Increasing intracellular fluorescence intensity indicated higher intracellular ROS activity [31].

#### DAPI Dye investigation of nuclear condensation

Using the fluorescent nuclear dye DAPI, the apoptotic impact of 6-gingerol was investigated [32]. As previously stated, the PC-3 cells were cultured and treated for 24 hours with efficacious concentrations of 6-G. After washing with water, 4% paraformaldehyde was used and it fixed the cells in 10 minutes. The cells were then stained with DAPI after being permeabilized with permeabilizing buffer (3% paraformaldehyde and 0.5% Triton X-100). Using a fluorescence microscope, pictures were collected and cell counts were calculated.

#### Investigation of mitochondrial membrane potential loss

MitoTracker dyes passively diffuse and accumulate in the mitochondria of live cells, it is widely used to evaluate MMP loss which is seen during apoptosis [33]. PC-3 in 24-well plates were treated with efficacious dosages of 6-G of 60 µM, 80 µM, 100 µM, and 120 µM. Following 24 hours, the treated cells were rinsed with PBS and stained with MitoTracker CMXRos dye for 30 minutes at 37^o^C in the dark. The images were captured using an inverted fluorescence microscope (Nikon ECLIPSE Ti-S, Japan), and the mitochondrial depolarization patterns of cells were evaluated using the imaging program NIS-Elements F 4.00.00.

#### Dual staining with Acridine Orange (AO) and Propidium Iodide (PI)

The number of live and dead cells was determined using a combination of acridine orange and propidium iodide labeling. As previously stated, the seeded PC-3 cells were treated for 24 hours with efficacious concentrations of 6-G. PBS was used to clean the media once it was dumped. A volume of 300 µL of 100µM AO and PI (1:1) was applied to PC-3 cells and incubated for 15 minutes at RT (room temperature) in the dark. The dye was then removed, and the cells were washed twice with PBS. The photographs were taken with a fluorescence microscope (Nikon ECLIPSE Ti-S, Japan) [34].

#### Assay for wound healing

The wound healing assay was used to evaluate the migration of PCa cells. PC-3 cells were cultivated in a 24-well plate and grown for 24 hours. To remove unattached cells, a scratch was produced in a single line with a sterile micropipette tip (200 µL) and washed with PBS. Cells were given effective dosages of 6-Gingerol of 60 µM, 80 µM, 100 µM, and 120 µM. Following 24 hours of exposure, each scratch was visualized and photos were captured at various concentrations [35].

#### Cell cycle phase distribution analysis by flow cytometry

Flow cytometry was done to determine cellular DNA contents at different cell cycle stages [38]. PC-3 cells were cultured with a density of 2x10^6^ cells/ml and were exposed to three effective doses *viz.* 80 µM, 100 µM, and 120 µM of 6-G. After 24h of exposure of 6-Gingerol to PC-3 cells, cells were harvested and washed with cold PBS. Further cells were fixed in 70% ethanol overnight, then 10 mg/mL of RNase A was incubated. After fixing cells, staining was done using PI dye (propidium iodide), followed by incubation in the dark at RT (30 mins.). The PI fluorescence of each nucleus was stained with PI dye and flow cytometry was done (FACS Calibur, Becton Dickinson, USA). BD FACSTM Software 1.2.0.87 was used to analyze data, and the result was expressed in percentage of the total number of cells in each cell cycle phase.

## Results and discussion

### Molecular docking simulations

#### Protein structure refinement

The crystal structure of human ERβ and AR proteins was carefully chosen for molecular docking investigation from PDB (http://www.rcsb.org/pdb/home/home.do). BDS Visualizer was used to display 3D structure and delete water, ions, hetatm, and ligand. Then, the obtained structure of target proteins was further subjected to energy minimized with the help of the YASARA server. 3Drefine servers were also used to more accurately refine the structure of receptor protein (Table 1).

**Table 1:** Details of refinement and energy minimized of receptor proteins by 3Drefine and YASARA server

Receptor	3Drefine server				Minimization (YASAR server)			
	3Drefine Score	MolProbity	RMSD(Å)	RWPlus	Start (kj/mol)	Score	End (kj/mol)	Score
ERβ	8310.53	1.389	0.252	-54319.69	673614.7	-0.79	-132953.4	0.60
AR	10085.3	1.574	0.242	-63571.34	-123285.4	0.22	-155440.2	0.97

#### Suitable pockets finding

**Fig. 2:**
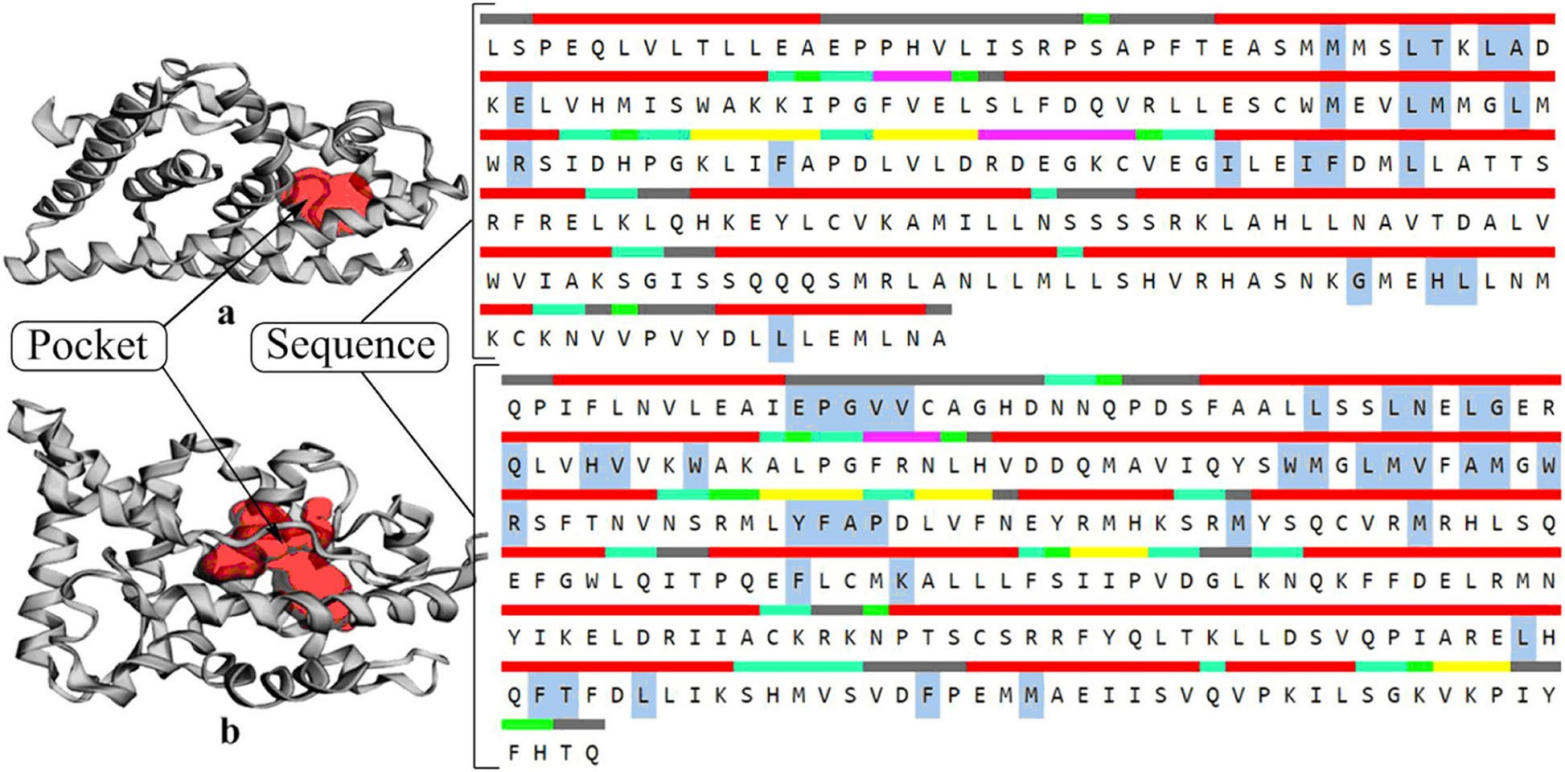
Cartoon structure of proteins (**a**) human estrogen receptor beta (Erβ); (**b**) androgen receptor (AR) with the solid red color pocket site; right-hand side represents a sequence of amino acid residues (Chain A) with highlighted boxes representing the amino acid residues present in the binding site or pocket

CASTp v3.0 server was utilized for the recognition of surface-accessible pockets and interior inaccessible cavities in target proteins. This server is quickly recognizing the main pocket site of the receptor protein along with the information on the pocket area, volume, and amino acid residues. The main pocket of human ERβ and AR proteins has an area of 186.427 and 356.797 with a volume of 117.855 and 190.024 (Table 2 & Fig. 2).

**Table 2:** Details of the pocket of receptor proteins

Receptor	Area	Volume	Sequence Length	Gene	Pocket amino acid residues
ERβ	186.427	117.855	241	ESR2	MET295, LEU298, THR299, LEU301, ALA302, GLU305, MET336, LEU339, MET340, LEU343, ARG346, PHE356, ILE373, PHE377, LEU380, GLY472, HIS475, LEU476, LEU491
AR	356.797	190.024	266	AR	GLU681, PRO682, GLY683, VAL684, VAL685, VAL685, LEU701, LEU704, ASN705, LEU707, GLY708, GLN711, HIS714, VAL715, TRP718, TRP741, MET742, LEU744, MET745, VAL746, VAL746, ALA748, MET749, TRP751, ARG752, TYR763, PHE764, ALA765, PRO766, MET780, MET787, PHE804, LYS808, LEU873, PHE876, THR877, LEU880, PHE891, MET895

#### Molecular docking simulation of human ERβ and AR

**Fig. 3:**
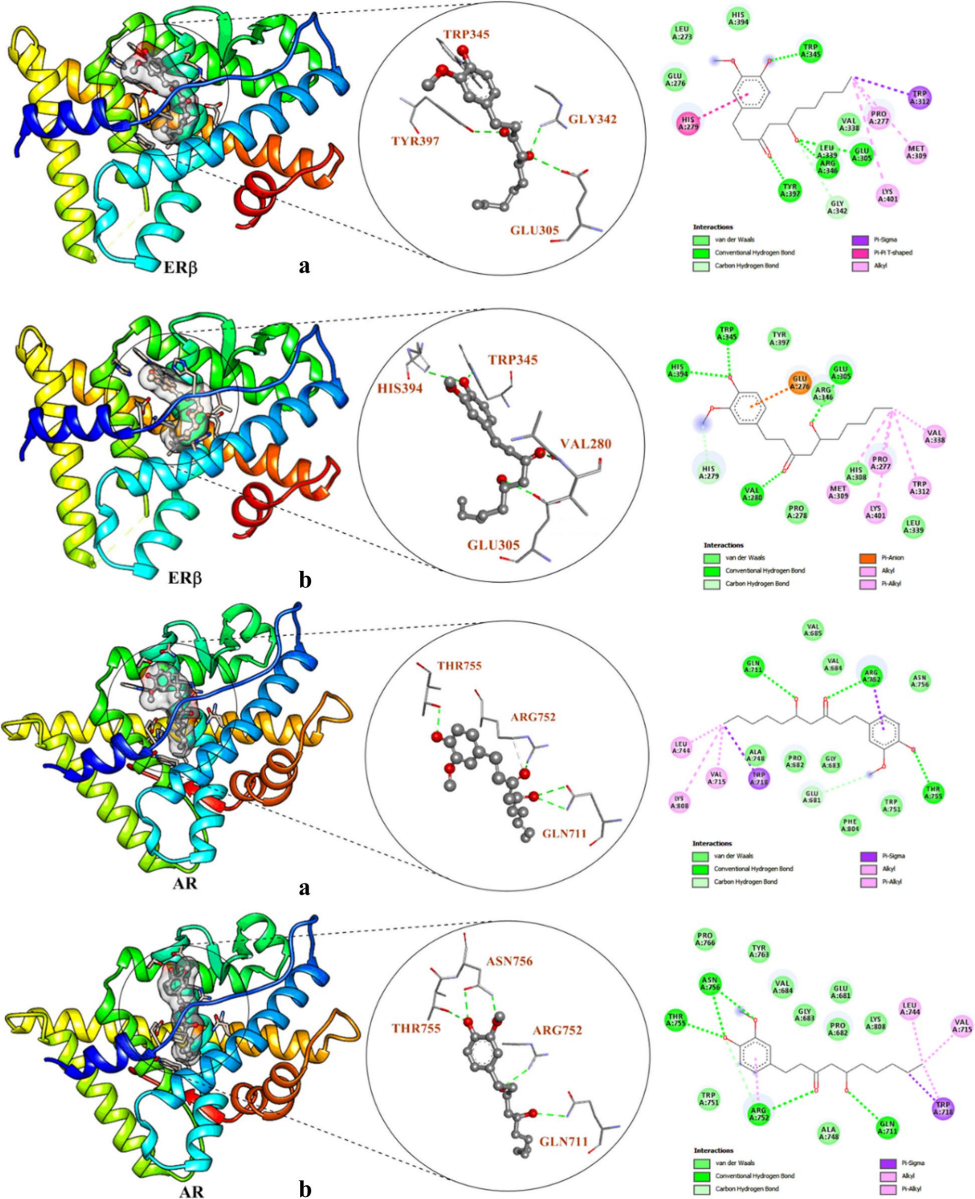
Showing 2D and 3D schematic representation of docking interactions of 6-Gingerol (**a**) Non-optimized (**b**) Optimized) with the receptor proteins human estrogen receptor beta (Erβ) and androgen receptor (AR)

Human ERβ and AR protein crystal structures were considered for molecular docking studies. While the crystal structure of 6-G was also obtained from PubChem and directly use for the docking evaluation with the help of iGEMDOCK. In this study, Bicalutamide was used as the control drug and four physiological ligands (2-(4-Hydroxy-phenyl)benzofuran-5-OL, 2,3-Dihydroxy-1,4-dithiobutane, Glycerol, and Testosterone) was also use as a reference for further analysis. Additionally, the 3D structure of 6-G was subjected to energy minimization with the help of MMFF94 for comparative docking evaluation. The binding interaction profile of 6-G was obtained in the form of the total energy a combination of VDW, H-Bond, and electrostatic energies. 6-G shows a good interaction profile against both the targets with − 105.05 and − 102.06 kcal/mol for non-optimized structure, while − 110.22 and − 103.46 kcal/mol for optimized structure (Table 3). The best docking poses display the interaction of 6-G with the same pocket amino acid residues (Fig. 3). The most interesting amino acid residues of ERβ are PRO277, PRO278, HIS279, VAL280, GLU305, HIS308, MET309, TRP312, VAL338 LEU339, TRP345, ARG346, HIS394, TYR397 and LYS401, respectively. While, GLU681, PRO682, GLY683, VAL684, GLN711, VAL715, TRP718, LEU744, ALA748, TRP751, ARG752, THR755, ASN756, TYR763, PRO766 and LYS808, respectively are interesting residues of AR. The 6-G non-optimized structure shows five H-bonding with residues of ERβ such as GLU305, GLY342, TRP345, ARG346, and TYR397, respectively along with the distance 2.86, 3.39, 2.99, 2.61 and 2.71 Å, respectively. while the optimized structure displays four H-bonding with residues of ERβ VAL280, GLU305, TRP345, and HIS394, respectively along with the distance 2.91, 2.95, 2.96 and 3.25 Å, respectively. Similarly, the non-optimized structure of 6-G also interacted with the AR through five H-bonding through three residues such as GLN711 (2.98 and 3.06 Å), ARG752 (2.87 and 3.20 Å) and THR755 (2.57 Å), respectively and optimized structure display five H-bonding interactions along with the four residues GLN711 (3.00 Å), ARG752 (3.05 Å), THR755 (2.74 Å) and ASN756 (2.61 and 2.70 Å), respectively. Therefore, the non-optimized structure shows more close interaction with the pocket residues as compared to the optimized structure with quite similar binding efficiency and H-bonding proficiency. The outcome was also compared to the control drug (Bicalutamide) as well as four physiological ligands and found that the control showed more binding efficiency (-119.76 kcal/mol) concerning ERβ as compared to 6-G (-103.46 kcal/mol), while physiological ligands depict lower potential between − 60.99 to -92.81 kcal/mol. Against the AR, the control drug showed slightly lower binding affinity (-107.67 kcal/mol) than the 6-G (-110.22 kcal/mol), and comparison of binding affinity (-58.69 to -92.48 kcal/mol) with physiological ligands also display their very effective binding potential. Therefore, from binding affinity evaluation, it is very clear that 6-G depicts their binding potential against AR (Table 3).

**Table 3:** iGEMDOCK molecular docking results along with the interacting amino acid residues

	Receptor	TE^**a**^	VDW^**b**^	HB^**c**^	Aver Con Pair	Amino acid residues		
						HB^**c**^	VDW^**b**^	Pi-Pi, Pi-Other
6-Gingerol (Non-optimized)	ERβ	-103.46	-84.46	-19	26.52	GLU305, TRP345, ARG346, TYR397	LEU273, GLU276, VAL338, LEU339, GLY342, HIS394	PRO277, HIS279, MET309, LYS401
6-Gingerol (Optimized)		-102.06	-80.86	-21.20	25.71	HIS279, VAL280, GLU305, TRP345, HIS394	PRO278, HIS308, LEU339, ARG346, TYR397	PRO277, MET309, TRP312, VAL338, LYS401
2-(4-Hydroxy-phenyl)benz of uran-5-OL		-92.81	-74.95	-17.8678	27.35	GLU305, LEU339, ARG346, GLY472, HIS 475	MET295, LEU301, ALA302, ILE373	LEU298, MET336, MET340, LEU343, PHE356
2,3-Dihydroxy-1,4-dithiobutane		-63.20	-41.15	-22.05	33	PRO278, VAL280, PHE356	PRO277, LEU301, GLU305, ARG346, ALA357, PRO358	HIS279
Glycerol		-60.99	-34.39	-26.60	37.67	GLU337, LEU462, ARG466	ILE404, ASN407, SER408, VAL465	-
Testosterone		-95.48	-88.48	-7.00	26.76	ARG346, HIS475	MET295, LEU301, GLU305, ILE373, LEU380, GLY472, ILE373, MET479	LEU298, ALA302, MET336, LEU339, MET340, PHE356, ILE376, LEU476
Bicalutamide		-119.76	-102.65	-17.10	26.069	GLU276, HIS279, TYR397	PRO278, LEU301, TRP345, ALA357, PRO258, HIS394, LYS401	LEU273, PRO277, VAL280, GLU305, PHE356
6-Gingerol (Non-optimized)	AR	-110.22	-98.27	-11.95	31.24	GLU681, GLN711, ARG752, THR755	GLY683, PRO682, VAL684, VAL685, ALA748, TRP751, ASN756, PHE804	VAL715, TRP718, LEU744, LYS808
6-Gingerol (Optimized)		-105.05	-87.49	-17.56	28.29	GLN711, ARG752, THR755, ASN756	GLU681, PRO682, GLY683, VAL684, ALA748, TRP751, TYR763, PRO766, LYS808	VAL715, TRP718, LEU744
2-(4-Hydroxy-phenyl)benzofuran-5-OL		-87.29	-83.41	-3.88	29.82	GLN711	GLU681, GLY683, VAL684, VAL685, LEU744, MET745	ARG752, LYS808
2,3-Dihydroxy-1,4-dithiobutane		-65.02	-42.70	-22.32	33.88	GLY683, VAL685, GLN711, PHE764	VAL684, LEU707, ARG752, ALA765, PRO766	TYR763
Glycerol		-58.69	-36.34	-22.35	42.50	TYR739, SER740, GLY743, LEU744, LEU811, GLN867	MET742, SER814, ILE815, ALA870, HIS874	-
Testosterone		-92.48	-83.98	-8.50	26.10	ASN705, GLN711, ARG752, THR877	LEU701, LEU707, GLY708, VAL746, MET749, PHE876, LEU880, PHE891, MET895	LEU704, TRP741, MET742, MET745, PHE764, MET780, LEU873
Bicalutamide		-107.67	-81.2454	-26.425	21.1379	ARG846, ARG855	TRP751, PHE754, PHE794, GLY795, LEU797, THR800, PRO801, GLU803, LYS845, ARG854, GLN858	TRP796, GLN798, ILE799

^a^TE (Kcal/Mol) = total binding energy

^b^VDW (Kcal/Mol) = Van Der Waals force energy

^c^HB (Kcal/Mol) = Hydrogen bond energy

#### IC_50_ calculation

Table 4 shows the IC_50_ values of 6-G in terms of binding energies computed using AutoDock. The obtained results exhibited that the calculated IC_50_ values for 6-G were in a range of 29.78–1.81 mM for ERβ, while 23.84 mM-131.41 µM for AR. Here, two types of docking (targeted and blank) were evaluated and found that targeted docking displays more potential for IC_50_ value as compared to blank docking. Based on the above finding, the most probable inhibitory target of 6-G is AR, and display a 131.41 µM value, which is much higher as compared to the 1.81 mM value against the ERβ. Therefore, the most suitable target of 6-G is AR. The obtained results of IC_50_ value were also compared with the control drug (Bicalutamide) and found that 6-G showed slightly lower potential against both receptor proteins (Table 4).

**Table 4:** Calculated binding energies (kcal/mol) and IC_50_ value

6-Gingerol	Receptor	Center coordinates of Grid box (Center)			Space sizes of Grid box (Dimension)			IC_50_ value (mM)	Binding energies (kcal/mol)
		x	y	z	x	y	z		
Non-optimized	ERβ	19.42	34.00	38.07	26	26	26	1.81	-3.74
		19.42	34.00	38.07	40	40	40	13.38	-2.56
Optimized		19.42	34.00	38.07	26	26	26	29.78	-2.08
		19.42	34.00	38.07	40	40	40	24.30	-2.20
Bicalutamide		19.42	34.00	38.07	26	26	26	83.62^a^	-5.56
		19.42	34.00	38.07	40	40	40	680.40^a^	-4.32
Non-optimized	AR	20.38	5.33	11.21	26	26	26	131.41^a^	-5.30
		20.38	5.33	11.21	40	40	40	7.59	-2.89
Optimized		20.38	5.33	11.21	26	26	26	23.84	-2.21
		20.38	5.33	11.21	40	40	40	10.58	-2.70
Bicalutamide		20.38	5.33	11.21	26	26	26	700.33^a^	-4.3
		20.38	5.33	11.21	40	40	40	81.00*	-5.58

^a^µM

#### HOMO-LUMO analysis

**Fig. 4:**
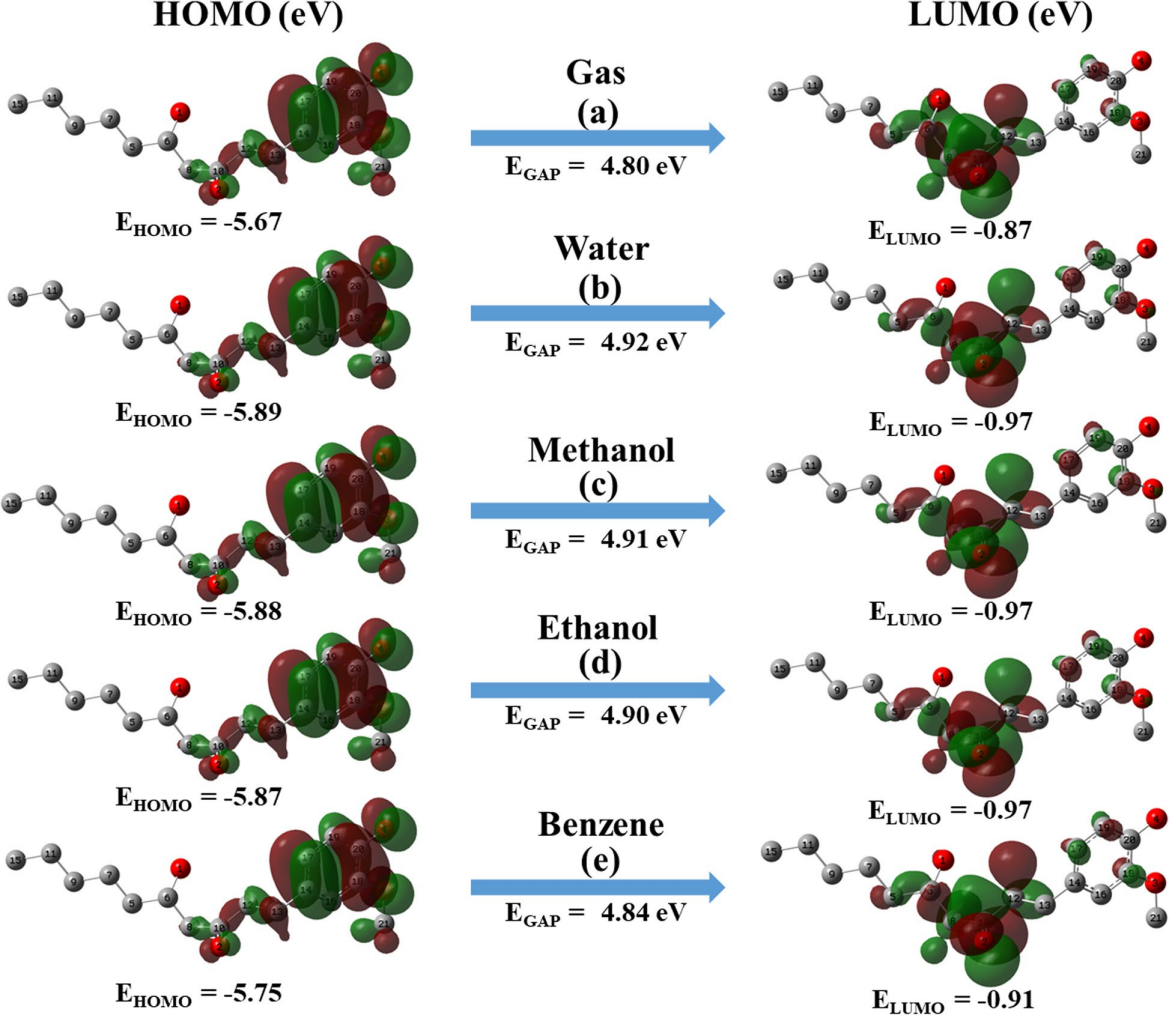
Atomic orbital composition of the HOMO, LUMO, and their E_GAP_ obtained from TD-DFT/B3LYP/6-311++G(2d,2p) level at gas and solvent phase

To evaluate the chemical stability and electronic properties of the 6-G the E_HOMO_, E_LUMO,_ and their E_GAP_ were calculated with the help of DFT/B3LYP/6-311G++(2d,2p), which is essential for the biological activity. During the chemical reaction, HOMO and LUMO perform a significant character in charge transfer among orbitals. The calculated values of the E_HOMO_, E_LUMO_, their E_GAP_ are displayed in Fig. 4. The negative charge is shown by the red color, whereas the positive charge is indicated by the green color. The HOMO-LUMO band gap of the 6-gingerol is calculated as 4.80, 4.92, 4.91, 4.90, and 4.84 eV in the gas, water, methanol, ethanol, and benzene phases, respectively.

As the molecule phase changes, the energy gap value changes by ~ 0.10 eV, and the smaller values of the HOMO-LUMO energy gap value suggest greater ease of electron transfers and reactivity. The softness of the molecule increases and the hardness decreases when compared to the gas phase molecule, so the molecule in the ethanol phase is more reactive. Soft molecules are typically more reactive and involve significant electron transfers, such as nucleophilic attacks.

### UV-visible analysis

**Fig. 5:**
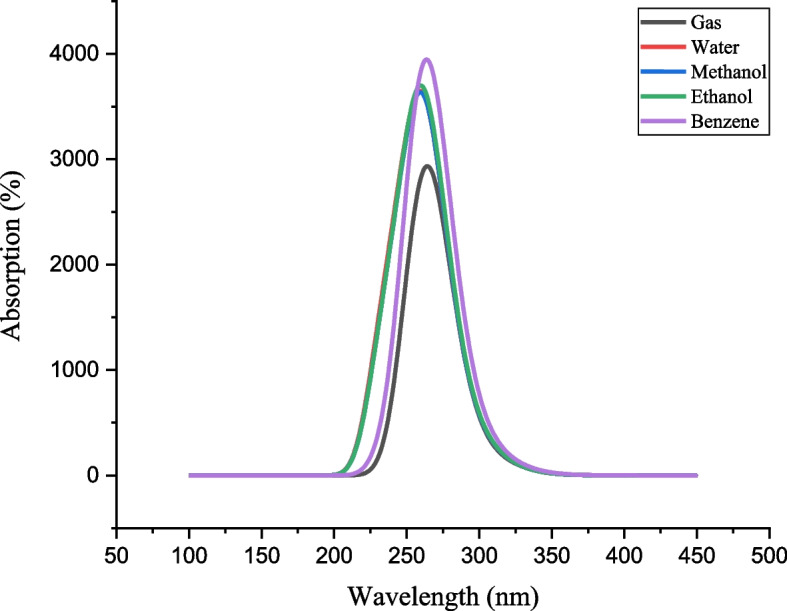
Comparison between the theoretical UV-Vis spectra (using the TD-DFT/B3LYP method with different solvation and gas effects) for 6-Gingerol

The properties of a molecular system in its excited state are examined by the TD-DFT/B3LYP/6-311G++(2d,2p). Figure 5 displays the combined theoretical UV-visible absorption spectra of the 6-G in the gas, water, methanol, ethanol, and benzene phases, respectively [44]. Table 5 lists the excited energies, oscillator strengths, and absorption spectra. Absorption peaks at 279 nm in the experimental UV spectra in the solvent (80% methanol) phase are associated with π→π* transitions [45]. The theoretical absorption peaks were obtained in gas and four different solvent phases (water, methanol, ethanol, and benzene). The computed absorption wavelength (λmax) is found to be 304, 278, and 264 nm for the gas, 297, 271, and 261 nm for the water, 297, 271, and 261 nm for the methanol, 298, 272, and 261 nm for the ethanol, and 302, 276, and 263 nm for the benzene. Among which, the λmax of the gas phase highly correlated and the methanol and ethanol phases likewise correlate with the experimental ones.

**Table 5:** Electronic properties of 6-Gingerol were calculated using the TD-DFT/B3LYP/6-311G++ (2d, 2p) basis set

S. No			λ_max_	Energy (eV)	Oscillating strength (f)	Assignments for major transitions	Major contributions (> 10%)
**1**	**Gas**		304.41	4.0729	0.0031	π→π*	H→L (52.63)
			278.45	4.4537	0.0021	π→π*	H→L (45.74)
			264.19	4.6930	0.0677	π→π*	H→L-2 (76.12)
**2**	**Solvents**	**Water**	297.62	4.1659	0.0051	π→π*	H→L (55.87)
			271.57	4.5655	0.0052	π→π*	H→L (42.11)
			261.39	4.7433	0.0763	π→π*	H→L-1 (81.64)
**3**		**Methanol**	297.89	4.1620	0.0051	π→π*	H→L (55.97)
			271.84	4.5609	0.0050	π→π*	H→L (42.06)
			261.52	4.7409	0.0764	π→π*	H→L-1 (81.78)
**4**		**Ethanol**	298.05	4.1598	0.0051	π→π*	H→L (56.00)
			272.01	4.5582	0.0052	π→π*	H→L (42.03)
			261.65	4.7385	0.0779	π→π*	H→L-1 (81.91)
**5**		**Benzene**	302.05	4.1047	0.0048	π→π*	H→L (55.65)
			276.03	4.4917	0.0048	π→π*	H→L (42.69)
			263.95	4.6972	0.0875	π→π*	H→L-1 (67.63)

#### Molecular descriptor and drug-likeness calculations

The Ro5-based Molinspiration server was frequently utilised in the prediction of molecular descriptors and drug-likeliness properties. Here it is also utilized to evaluate the properties of 6-G. These properties are important in the formation and development of chemical species' bioactivity. Molinspiration open server was commonly used to find the parameter including topological polar surface area (TPSA), Octanol-water partition coefficient (LogP), molecular weight (MW), nON, nNHOH, and drug-likeness. LogP, as a measure of molecule hydrophobicity, plays an important role in quantitative structure-activity relationship assessment and rational drug design. It is particularly useful for assessing drug absorption, bioavailability, hydrophobic drug-receptor interactions, metabolism, and toxicity. TPSA is another significant parameter for the observation of drug transport properties and is defined as a sum of surfaces of polar atoms (typically oxygen, nitrogen, and attached hydrogen) in a chemical entity. MW represents the abortion and transportation of the chemical species. nON and nNHOH are represents the interaction profile of the chemical species. A higher number of H-bond donors and acceptors in the chemical species restrict their absorption and assimilation in the intestine. Since then, the absorption percentage has been determined with the aid of the TPSA value using the formula: Absorption (%) = 109 − [0.345 × TPSA] as shown in Table 6.

### Result about is good for drug-likeness or not

**Table 6:** The molecular descriptor, Bioactivity radar, and bioactivity score of 6-Gingerol

Molecular descriptor	
Paraments	Values
LogP	3.22
Topo polar surface area (TPSA)	66.76
Number of hydrogen bond donors (nON)	4
Number of hydrogen bond acceptors (nNHOH)	2
Molecular weight (MW)	294.39
Number atom (nAtom)	21
Number rotatable (nRotab)	10
Volume	295.61
Absorption percentage (%)	85.97
**Bioactivity radar**	

**Bioactivity score**	
GPCR ligands	0.16
Ion channel modulators (ICM)	0.04
Kinase inhibitors (KI)	-0.33
Nuclear receptors ligand (NRL)	0.20
Protease inhibitor (PI)	0.15
Enzyme inhibitor (EI)	0.38

### Bioactivity score

The bioactivity of 6-G was also determined by estimating the activity score against several targets such as GPCR ligand, NRL, ICM, PI, EI and KI. The molinspiration bioactivity score was utilized to check all the activity parameters. The 6-G fulfils the rations of Drug-likeness and bioactivity. The 6-G shows good potential against the GPCR ligand as well as the Nuclear receptors ligand. While it displays outstanding results as an enzyme inhibitor. The obtained results specify that 6-G has a good bioactivity score and was offered in Table 6. The bioactivity score findings show 6-G efficiency in order: Enzyme inhibition > Nuclear receptor-ligand > GPCR ligand > Protease inhibitor > Ion channel modulation > Kinase inhibitor. A chemical species possessing a bioactivity score greater than 0.00 is most probable to display significant biological activities, whereas values − 0.50 to 0.00 are projected to be mild active and when the score is smaller than − 0.50 it is alleged to be inactive. The results disclose that the biological actions of 6-G might include various mechanisms in the presence of interactions with all the biological targets. The 6-G displays considerable attention and represents better to good interactions with ICM, PI, GPCR ligands, NRL, and EI while showing moderate interaction with KI (Table 6).

#### ADMET profile

The ADMET profile of 6-G was evaluated with the help of admetSAR and various properties were calculated such as BBB penetration, HIA, Caco-2 cell permeability, and Ames test. admetSAR is an open-source repository with a molecular built-in user interface that supports SMILES and fragment-based search to query the database. It delivers the latest and comprehensive, curated data for various chemical species related to identified ADMET profiles. The information resulting from the admetSAR server exposed that the 6-G was shown well HIA. The HIA values suggest that the compound could be readily absorbed from the gastrointestinal system following oral administration. The observed value of BBB penetrability critically relates to the chemical structures of 6-G, a less polar chemical structure was calculated to penetrate BBB. The value of P-glycoprotein (P-gp) was also calculated and obtained results display as a P-gp substrate and P-gp inhibitor, 6-G is evaluated as a P-gp substrate. The mutagenicity of 6-G was also evaluated in terms of an ames toxicity test and obtained results reveal that the compound is non-mutagenic. Carcinogenicity (binary and trinary) offer information about the carcinogenic profile of the compound, obtained results show 6-G is non-carcinogenic (Table 7).

**Table 7:** ADME prediction of 6-Gingerol based on admetSAR server

Absorption				
**Parameters**		Value	Probability	Finding
Human intestinal absorption (HIA)		+	0.9924	Yes
Human oral bioavailability (HOB)		-	0.7714	No
Caco-2 permeability		+	0.5943	Yes
Log Kp (skin permeation)		-	-6.14 cm/s	-
**Distribution**				
Blood-brain barrier penetration (BBB)		+	0.7879	Yes
Estrogen receptor binding (ERB)		+	0.8332	Yes
Androgen receptor binding (ARB)		-	0.5000	No
P-glycoprotein substrate		+	0.5562	Yes
P-glycoprotein inhibitor		-	0.8934	No
Plasma protein binding (PPB)		0.857	100%	Yes
**Metabolism**				
Cytochrome P450 (CYP450)	CYP3A4 substrate	+	0.5566	Yes
	CYP3A4 inhibition	-	0.5902	No
	CYP2C9 substrate	-	0.7974	No
	CYP2C9 inhibition	-	0.8278	No
	CYP2D6 substrate	+	0.3792	Yes
	CYP2D6 inhibition	-	0.7926	No
Pharmacokinetics transporters	OATP1B1 inhibitor	+	0.8982	Yes
	OATP1B3 inhibitor	+	0.9091	Yes
	BSEP inhibitor	+	0.7452	Yes
**Excretion and Toxicity**				
Organ toxicity	Human either-a-go-go-ralted gene (hERG) inhibition	+	0.7369	Yes
	Acute oral toxicity	2.29	kg/mol	-
Genomic toxicity	Ames mutagenesis	-	0.5700	No
	Carcinogenicity (binary)	-	0.7000	No
	Carcinogenicity (trinary)	Non-required	0.7188	No
Eco-toxicity	Crustacea aquatic toxicity	-	0.5185	No
	Biodegradation	-	0.6250	No
	Hepatotoxicity	-	0.8000	No
	Eye corrosion	-	0.9803	No
	Eye irritation	+	0.9328	Yes
	Honeybee toxicity	+	0.7611	Yes
	Fish aquatic toxicity	-	0.9799	Yes
	*Tetrahymena Pyriformis*	0.888	pIGC_50_ (ug/L)	-

#### BOILED-Egg’s model

**Fig. 6:**
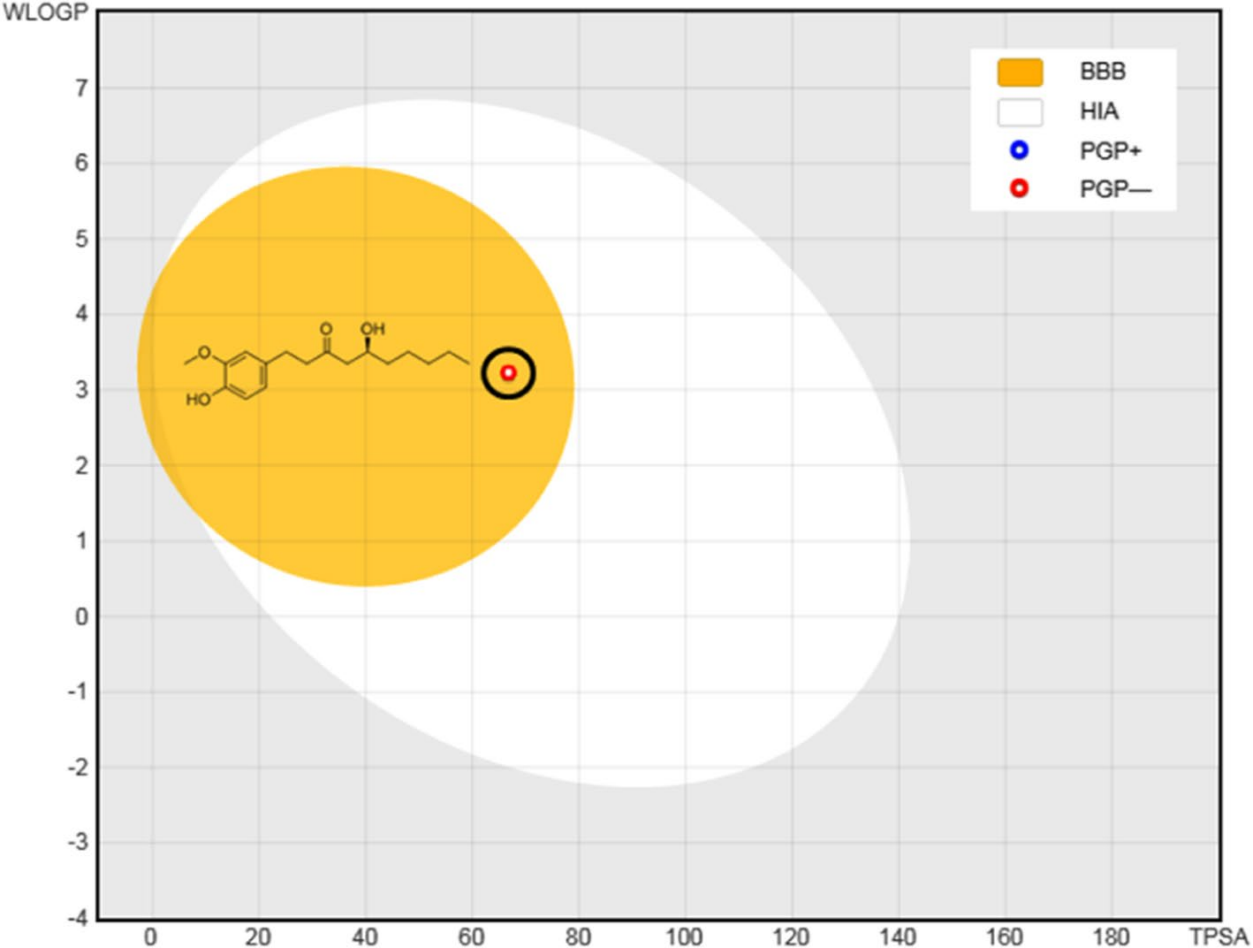
Displayed BOILED-Egg’s Model of 6-Gingerol

To more accurately predict the ADMET profile, BOILED-Egg’s Model was further used (Fig. 6). The red pointed site in BOILED-Egg’s yolk is molecules projected to passively cross the blood-brain barrier (BBB) and also absorbed by the gastrointestinal tract. The PGP + symbol represented by blue dots is for molecules to be crossed from the CNS by the P-glycoprotein, while the PGP- represented by red dots is for molecules calculated not to be collapsed from the CNS by the P-glycoprotein. The obtained finding displays that the 6-G fibbed inside the white ellipse and inside the BOILED-Egg’s yolk too.

### Biological

#### Inhibition of cell viability in PC-3 cells

**Fig. 7:**
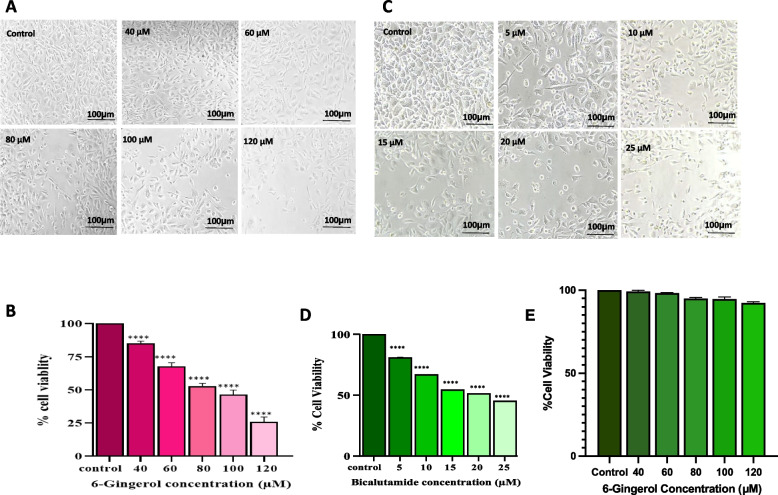
*In vitro *cell viability activity of 6-Gingerol and Bicalutamide against human PCa PC-3 cells. **A** Live and dead PC-3 cells were evaluated morphologically treated with 40-120 µM concentration of 6-G (**B**) The viability of PC-3 cells measured in percentage by MTT assay at 24 h. Means ± SEM values are expressed of minimum three experiments independently, **p<0.01, ***P < 0.001, and ****p<0.0001 in comparison to their particular control.** 1 ***In vitro *cell viability activity of Bicalutamide against human PCa PC-3 cells. **C** PC-3 cells were evaluated morphologically with 5-25 µM concentration of Bicalutamide. **D** The viability of PC-3 cells measured in percentage by MTT assay at 24 h. Means ± SEM values are expressed of minimum three experiments independently, **p<0.01, ***P < 0.001, and ****p<0.0001 in comparison to their particular control. **2** *In vitro *cell viability activity of 6-Gingerol human HaCaT cells. **E** Graph showed the percent cell viability of HaCaT cells measured by a MTT assay at 24 h. Means ± SEM values are expressed of minimum three experiments independently, **P < 0.01, ***p<0.001 and ****p<0.0001 in comparison to their particular control

An inverted phase contrast microscope was used to observe PC-3 cells for morphological changes treated with selected concentrations of 6-G and control drug BT for 24 h (Fig. 7). The photomicrographs revealed altered shapes, cellular shrinkage, and surface detachment of 6- and BT treated cells as compared to untreated PC-3 cells. The 24 h of treatment of 6-Gingerol of different doses viz. 40, 60, 80, 100 and 120 µM and Bicalutamide of different doses viz. 5, 10, 15, 20 and 25 µM followed by incubation with MTT dye show a decrease in percent cell viability in a dose-dependent manner as shown in (Fig. 7A, Fig. 7.1C). The percent cell viability data showed that 40 µM, 60 µM, 80 µM, 100, and 120 µM doses of 6-Gingerol resulted in significant reductions in cell viability of approximately 85.33%, 68.00%, 53.00%, 46.66%, and 26.00% (*P* < 0.001) as compared to control (Fig. 7B). 5, 10, 15, 20 and 25 µM doses of BT result in a significant reduction in viability of cells to approximately 81.15%, 67.14%, 54.71%, 51.43%, and 45.41% as compared to control (Fig. 7.1D). 6-G and control drug BT inhibits cell proliferation in PC-3 cells and most cytotoxic doses were last three. IC50 value of 6-G was found to be at 100 µM concentration and control drug BT was at 20 µM concentration. Therefore, three optimum doses were selected (80, 100, and 120 µM) of 6-G for further studies. Thus, the data revealed that BT halts cellular growth effectively and also confirms that the compound 6-G shows similar effects in inhibiting cell proliferation of PC-3 cells.

The phase contrast inverted microscope was used to study the dose-dependent effect of compound 6-G on the cellular morphology of normal HaCaT cells (Nikon ECLIPSE Ti-S, Japan). The cells were cultivated overnight before being treated for 24 hours with various effective concentrations of tested compounds *viz.* 40 µM, 60 µM, 80 µM, 100 µM, 120 µM of 6-Gingerol. After 24 hours of exposure of 6-Gingerol against HaCaT, no toxicity was observed in the normal HaCaT cells. Graph showed the percent cell viability of HaCaT cells measured by a MTT assay at 24 h (Fig. 7.2E). 6-Gingerol show potent cytotoxicity against PC-3 cells but not on normal HaCaT cells as revealed by MTT cell viability assay data.

#### Gingerol generate ROS intracellularly in PC-3 cells

**Fig. 8:**
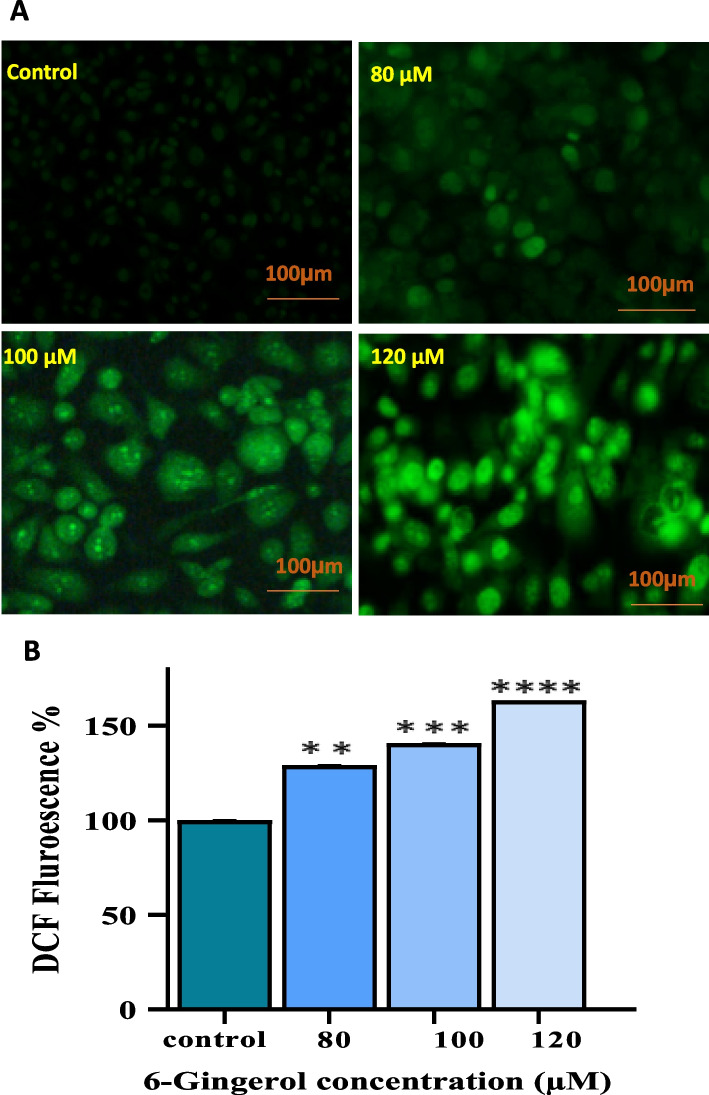
Intracellular ROS accumulation in PC-3 cells with DCFH-DA staining after 6-G treatment. **A** Photomicrograph showed ROS production intracellularly induced by 80, 100 and 120 µM of 6-Gingerol. Pictures were analysed phase contrast microscope. **B** Data are expressed numerically as fluorescence intensity percentage compared to control of PC-3 cells. Means ± SEM values are expressed of minimum three experiments independently, **P < 0.01, ***p<0.001 and ****p<0.0001 in comparison to their particular control

High intracellular ROS production can result in the oxidation of PC-3 cell macromolecules and subsequent cell injury. By increasing ROS production in a dose-dependent way, the intensity of DCFH-DA in PC-3 treated cells was found to increase (Fig. 8A). As shown in Fig. 8B, PC-3 cells treated with 6-Gingerol for 24 hours produced significantly more reactive oxygen species (ROS) than untreated cells by 29.13%, 40.70%, and 63.25% at 80, 100, and 120 µM, respectively. Thus, the results provide sufficient evidence that 6-G is responsible for inducing early apoptosis via many pathways via enhanced ROS production.

#### Gingerol causes change in nucleus with apoptosis within PC-3 cells

**Fig. 9:**
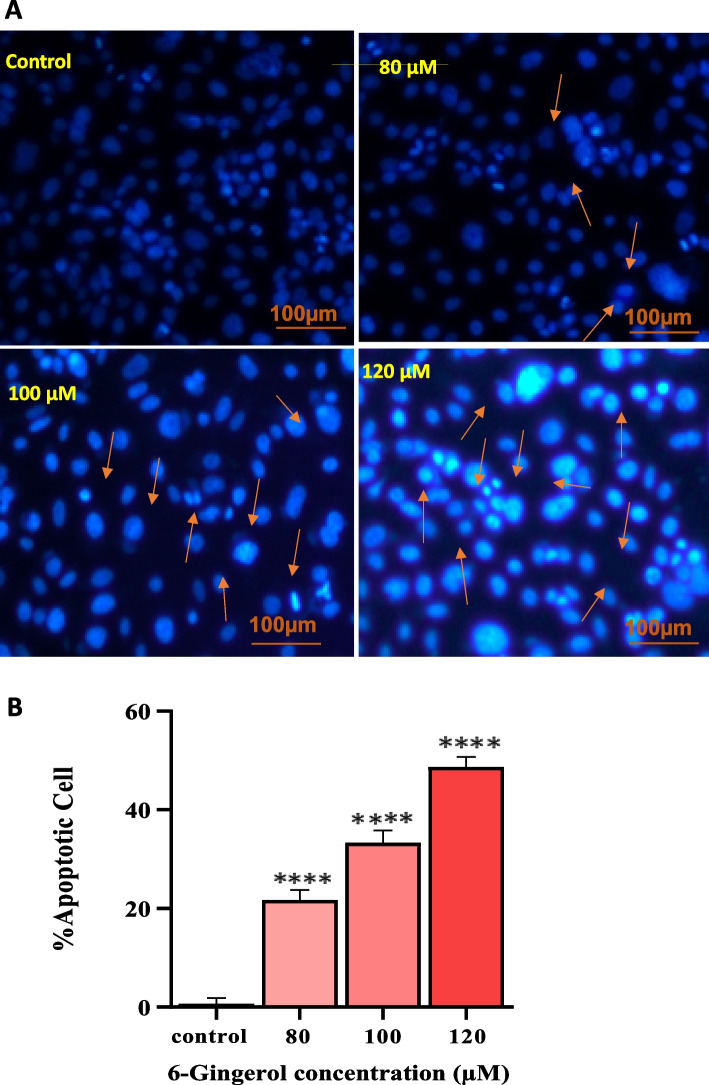
Nuclear condensation of PC-3 cells with DAPI staining after 6-G treatment. PC-3 cells were treated with 80, 100 and 120 µM of 6-G. **A** Pictures depicts nuclei as condensed and fragmented as captured by phase contrast fluorescence microscope. **B** Data expressed numerically as percent apoptotic cells compared to their control of PC-3 cells. Means ± SEM values are expressed of minimum three experiments independently, **P < 0.01, ***p<0.001 and ****p<0.0001 in comparison to their particular control

DAPI-based fluorescence microscopy revealed fragmented, condensed chromatin and nuclear apoptotic bodies in 6-G-treated cells stained with a fluorescent DAPI dye (Fig. 9A). In a concentration-dependent manner, 6-G induces apoptosis in PC-3 cells labeled with DAPI. The apoptotic cells percentage in PC-3 cells treated with 80 and 100 µM 6-G increased by roughly 21,66% and 33,333%, respectively, according to the quantitative data (Fig. 9B). Highest number of condensed chromatin was found in PC-3 cells treated with 120 µM, and the proportion of apoptotic cells increased to 48.66 percent. The results indicate that 6-G induces cell death *via* apoptosis in human PCa cells.

#### Gingerol modulates mitochondrial membrane potential in PC-3 cells

**Fig. 10:**
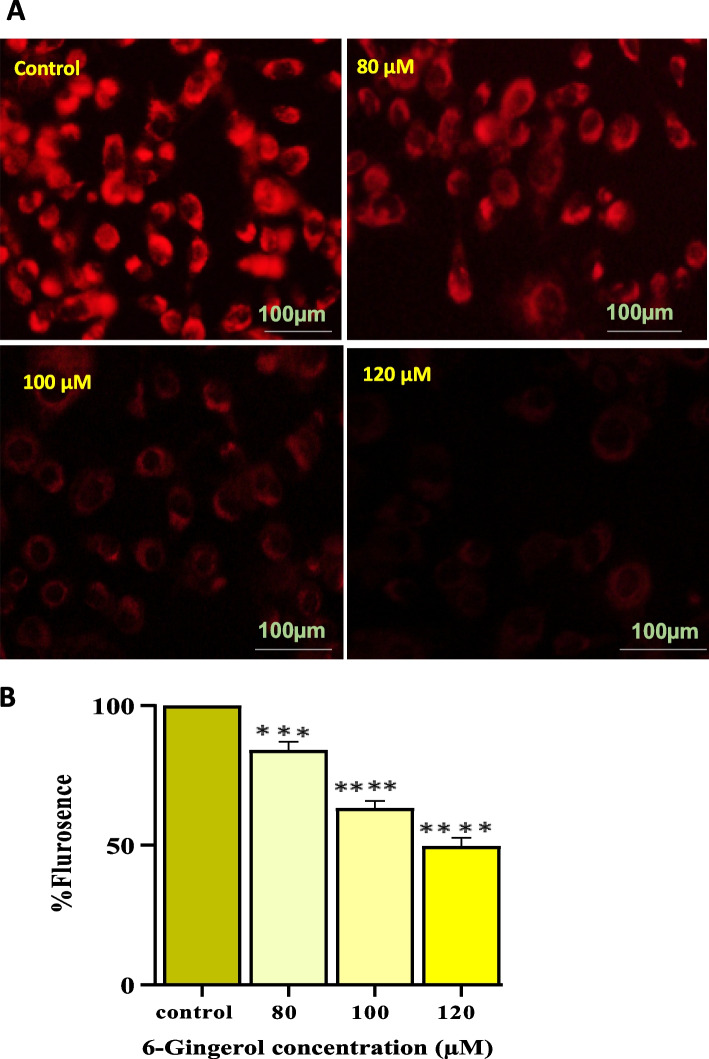
Photomicrographs showed PC-3 cells after staining with MitoTracker Red CMXRos dye after incubating for 24 h with concentrations of 80, 100 and 120 µM of 6-G. **A** Photomicrographs show a decrease in MMP (early event of apoptosis) with increasing concentrations of 6-G. **B** Data represented numerically as % Red fluorescence cells decreased with increase in doses of 6-Gingerol. Means ± SEM values are expressed of minimum three experiments independently, **P < 0.01, ***p<0.001 and ****p<0.0001 in comparison to their particular control

Increased green fluorescence as a result of MitoTracker Red CMXRos dye indicated a change in MMP. In a dose-dependent way, PC-3 cells treated with 80, 100, and 120 µM of 6-G exhibited a bright green fluorescence (Fig. 10A). As demonstrated by quantitative data, it rises from 9.33% in control cells to 84.0%, 63.3%, and 49.66% at 80 µM, 100 µM, and 120 µM concentrations, respectively (Fig. 10B). Hence, the results indicate that all concentrations of 6-G exhibit promising apoptotic potential and significantly trigger apoptosis in PC-3 cells.

#### Gingerol promotes apoptosis in PC-3 cells

**Fig. 11:**
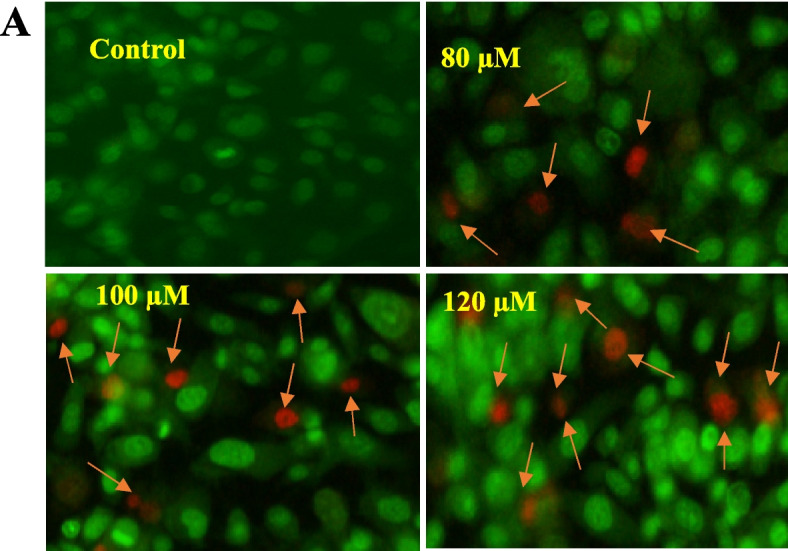
Photomicrographs showed PC-3 cells stained with AO/PI after 24 h incubation with concentrations of 80, 100 and 120 µM of 6-G. **A** Green and red fluorescence depicts viable and dead cells respectively**. **Photomicrographs show an increase in apoptosis with increasing concentrations of 6-G

Under a fluorescent microscope, the number of living and non-living cells was labeled and analyzed. Intact membranes emitted green fluorescence, indicating cell viability, whereas fractured membranes and dead cells emitted orange and red fluorescences, respectively (Fig. 11). Increasing doses of 6-G led to a substantial increase in the number of dead cells compared to the control group. Our data demonstrate that 6-G induces apoptosis in PCa PC-3 cells.

#### Gingerol suppresses the migration of PC-3 cells

**Fig. 12:**
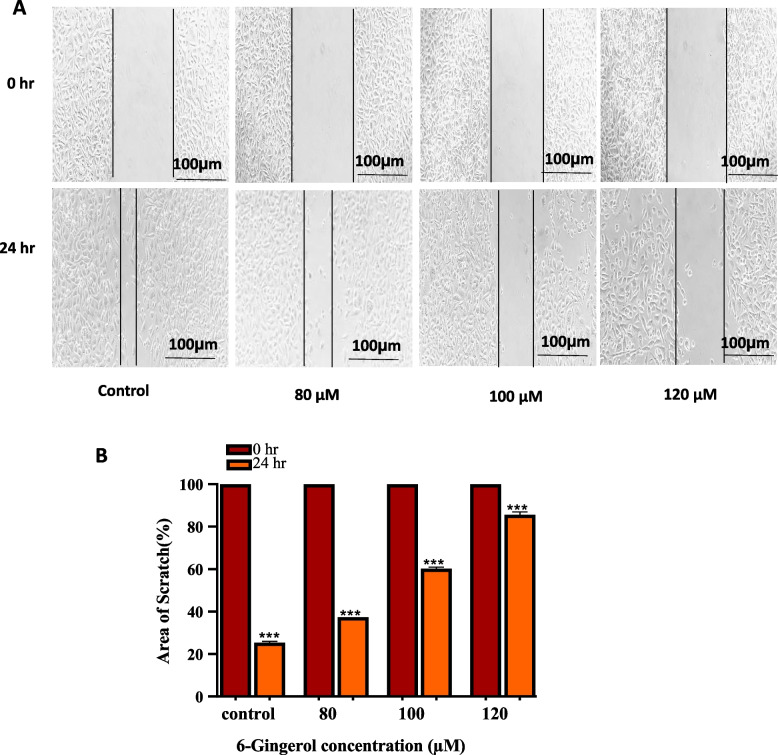
Wound healing assay to determine migration ability of PC-3 cells after 6-G treatment with concentrations of 80, 100 and 120 µM. 0 and 24 h time points were taken to capture images. **A** Photomicrographs show a reduction of migration and an increase in wound size with increasing concentrations of 6-G. **B** Data stated numerically as percent wound healing compared to their control of PC-3 cells. Means ± SEM values are expressed of minimum three experiments independently, **P < 0.01, ***p<0.001 and ****p<0.0001 in comparison to their particular control

The effect of 6-G on the migration of PC-3 cancer cells was evaluated using a wound-healing assay. At 80 and 100 µM concentrations of 6-Gingerol, migration ability was inhibited. At 120 µM of 6-Gingerol, the greatest decrease in migration and the most significant rise in wound size were seen due to an increase in cell mortality (Fig. 12). The untreated cancer cells exhibited normal migration, and the incision completely healed within twenty-four hours. The data displays 0h, control, and treated PC-3 cells.

#### Modulation of PC-3 cellular DNA content

**Fig. 13:**
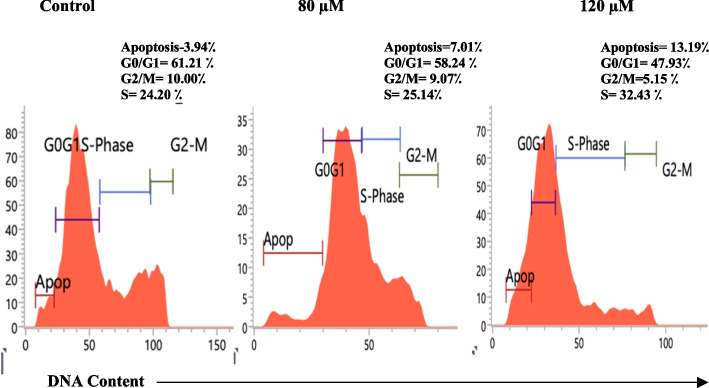
Effect of 6-G on Cell cycle kinetics. The pictorial representation of apoptosis and phase distribution of cells treated with 80 μM and 120 μM of 6-Gingerol

Analysis of phases of cell cycle with PC-3 cells DNA content at 80 µM and 120 µM of 6-G was done using flow cytometer. The percentage of dead cells was calculated by the number of sub-diploid cells in the various phases of the cell cycle histogram. According to the results, the number of apoptotic cells in untreated PC-3 cells was 3.94%. However, 6-Gingerol treatment changed the cell cycle progression and increased the apoptosis to 7.01% and 13.19% at 80 and 120 µM, respectively (Fig. 13). Untreated PC-3 cells in G0/G1 phase were 61.21%. PC-3 cells treated with 80 and 120 µM show cell cycle arrest of approximately 58.24% and 47.93% in the G0/G1 phase as compared to the control. The number of cells was less in the G2/M phase than in the control, which explains checkpoints at the G0/G1 phase reducing S phase DNA content in the cell cycle (Fig. 13).

## Conclusion

AR remains important in the progression of PCa thereby targeting AR pathway can be more effective therapeutic approach. *In silico* approach has become an inspiring procedure for the quick finding of possible inhibitors of a receptor protein in drug development. Using a combination of molecular docking, molecular descriptor (Lipinski rule), and predicted oral bioavailability, 6-Gingerol was shown to have a high calculated binding affinity in the active pocket of the receptor proteins. Druglikeness, bioactivity score, ADMET, and BOILED-Egg’s Model investigation were exposed to satisfactory results. The 6-G can be measured as a promising inhibitor of human AR with the IC_50_ value of 131.41µM as compared to human ERβ (IC_50_ value: 1.81mM), It is also correlated with the iGEMDOCK molecular docking results − 110.22 kcal/mol for ERβ and − 103.46 kcal/mol for AR. The result of the E_HOMO_, E_LUMO_, and E_GAP_, display that 6-G is more active towards biological actions. Thus, 6-G showed good affinity *in silico* observation and was further evaluated for *in vitro* analysis. *In vitro* study suggested that 6-G possesses anti-cancer activity by inducing cell apoptosis in human PCa PC-3 cells by affecting AR regulated DDR genes (*BRCA1, BRCA2*, and *ATM* ) which is involved in cell survival. 6-G leads to cell death by excessive ROS generation, nuclear fragmentation, mitochondrial membrane depolarization, cell cycle arrest and cell death without affecting the normal cell of the body. Therefore, present data confirm the potential of 6-G in regulating AR signalling pathway by promoting anti-cancerous activity and ability to inhibit the development and progression of PCa. Thus, it is crucial to pursue well-designed clinical trials to develop 6-G as an effective chemotherapeutic agent against PCa in the near future.

## Data Availability

The datasets used and/or analysed during the current study available from the corresponding author on reasonable request.

## Abbreviations

AR: Androgen receptor
6-G: 6-Gingerol
Bax: (B-cell lymphoma)‐associated X
Bid: B H3- i nteracting domain d eath agonist
Bcl-2: B-cell lymphoma 2
XIAP: X-linked inhibitor of apoptosis protein
BT: Bicalutamide
PDB: Protein Data Bank
ERα: Estrogen receptorα
ERβ: Estrogen receptorβ
MTT: 3-(4,5-dimethylthiazol-2-yl)-2,5-diphenyltetrazolium bromide
MMP: Matrix metalloproteinase
DAPI: Diamidino-2-phenylindole dihydrochloride
AO/PI: Acridine orange/ propidium iodide
CASTp: Computed Atlas of Surface Topography of proteins
MMFF94: Merck Molecular Force Field
LGA: Lamarckian genetic algorithm
DFT: Density functional theory
FMOs: Frontier molecular orbitals
HOMO: Highest occupied molecular orbital
LUMO: Lowest unoccupied molecular orbital
NBMO: Non-bonding molecular orbital
QCP: Quantum chemical parameter
ADMET: Absorption, distribution, metabolism, excretion and toxicity
PPB: Plasma protein binding
BBB: Blood-brain barrier
P-gp: P-glycoprotein
CNS: Central nervous system
HIA: Human intestinal absorption
GPCR: G-protein coupled receptor
ICM: Ion channel modulator
KI: Kinase inhibitor
NRL: Nuclear receptor ligand
PI: Protease inhibitor
TPSA: Topological polar surface area
LogP: Octanol-water partition coefficient
MW: Molecular weight
EI: Enzyme inhibitor
RPMI: Roswell Park Memorial Institute
FBS: Fetal bovine serum
DCFH-DA: 2′,7′-Dichlorofluorescein diacetate
DAPI: 4′,6-diamidino-2-phenylindole
DMSO: Dimethyl sulfoxide
PSA: Prostate-Specific Antigen
ROS: Reactive oxygen species
TNF-α: Tumor necrosis factor alpha gene
